# Supplementary material for: Macrophages Promote Tumor Cell Extravasation across an Endothelial Barrier through Thin Membranous Connections
Source: Cancers (Basel). 2023 Mar 31;15(7):2092. doi: 10.3390/cancers15072092 (PMC10093384; doi:10.3390/cancers15072092)
Supplement: Supplementary file 1 [file cancers-15-02092-s001.zip › cancers-2257486-supplementary.pdf]

A)

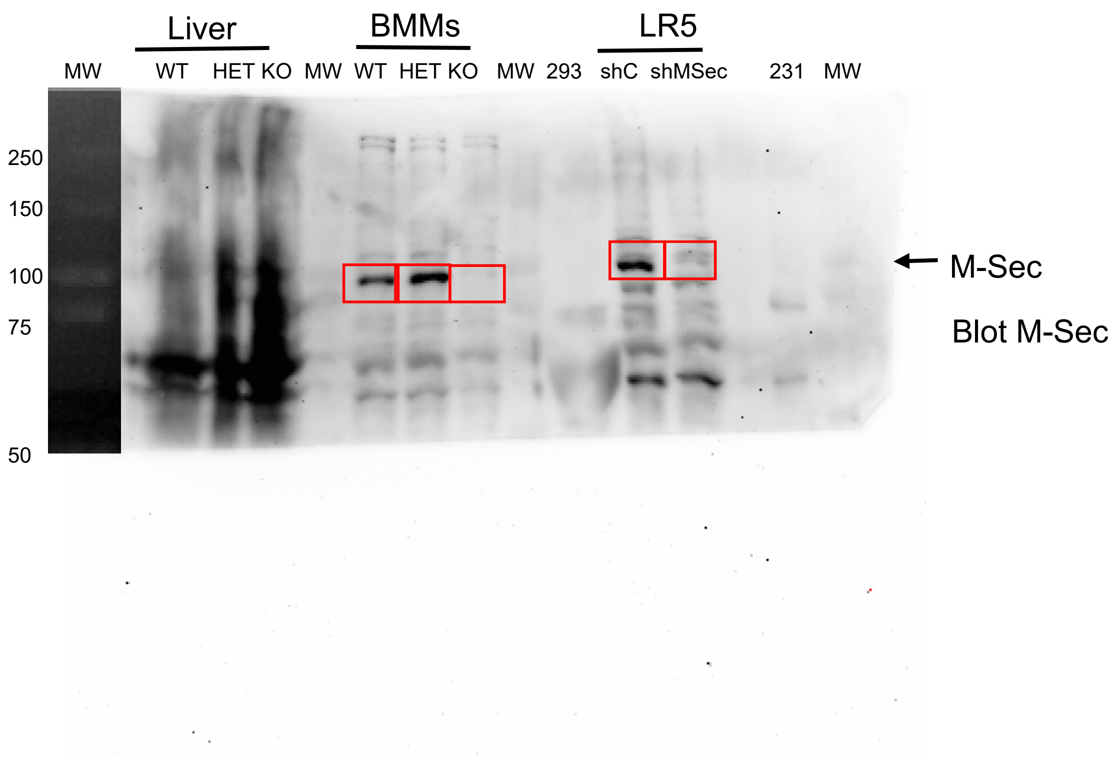

B)

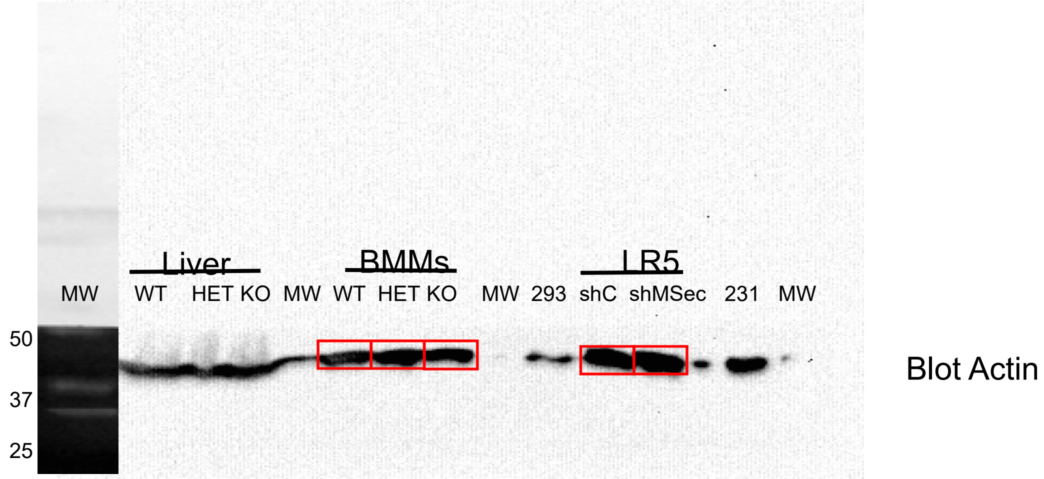

C)

	M-Sec	Actin	normalized	Ratio
BMM WT	459216	45721	10.04387481	
BMM Het	559854	46284	12.09605911	1.204322
BMM KO	186006	58862	3.160035337	0.314623
LR5/shCtrl	470107	71396	6.584500532	
LR5/shMSec	239035	77892	3.06880039	0.466064

Figure S1: Detection of M-Sec protein levels in the various macrophages used in this study (Supports figures 3C and 4A in the main text). Full western blots of whole cell lysates from mouse liver (purposely overloaded due to the reported low M-Sec levels) or bone marrow derived macrophages (BMMs) isolated from either WT, heterozygous (HET) or M-Sec KO (KO) mice, RAW/LR5 macrophages expressing either shRNA control (shC) or shRNA M-Sec (shMSec), and MDA-MB-231 (low M-Sec levels). The blot was cut at the 50kDa marker and probed separately using specific antibodies against M-Sec/TNFAIP2 (AbCam, ab91235) (upper) in A) and B) actin (Sigma) for a loading control (lower). Imaging and quantification of the blots was done using the Kodak Image Station 440 and software. C) For the cells used in this study, (Fig. 3C and 4A) the sum intensity of the boxed areas (red outline) were background subtracted. The M-Sec intensities were then normalized to the actin levels. The level of reduction is shown as a ratio to the WT BMMs or the LR5/shC levels.

A)

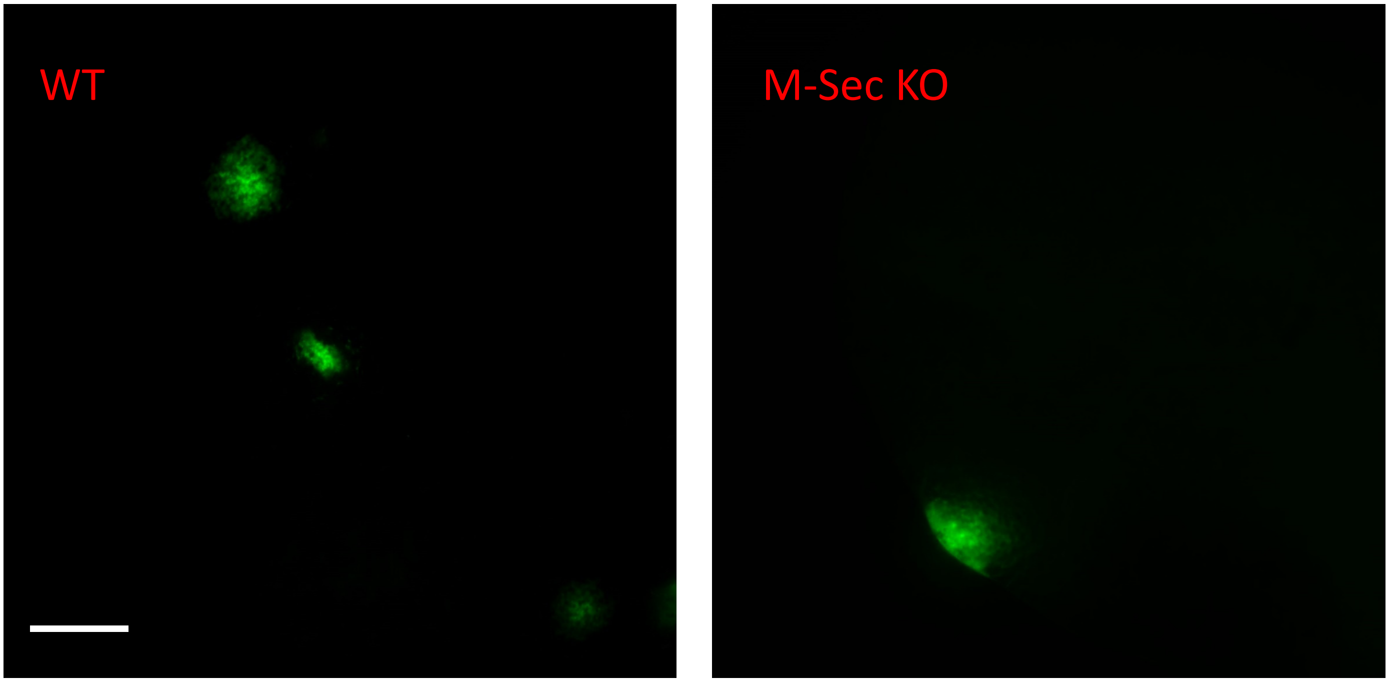

Figure S2: Widefield images of GFP-E0771 lung metastases in WT and M-Sec KO mice. Supports figure 4E of the main text. Uncropped images of lung metastases derived from E0771-GFP cells injected in the tail vein of either WT or M-Sec KO mice 7 days after tumor cell injection. Dissected lungs were imaged immediately on an Olympus IX71 microscope using a 5X long working distance lens, scale-bar 200 μm.
